# Formation of nanoflowers: Au and Ni silicide cores surrounded by SiO*_x_* branches

**DOI:** 10.3762/bjnano.14.14

**Published:** 2023-01-20

**Authors:** Feitao Li, Siyao Wan, Dong Wang, Peter Schaaf

**Affiliations:** 1 Chair Materials for Electrical Engineering and Electronics, Institute of Materials Science and Engineering and Institute of Micro- and Nanotechnologies MacroNano, TU Ilmenau, Gustav-Kirchhoff-Straße 5, 98693 Ilmenau, Germany

**Keywords:** Au/Ni bilayers, dewetting, vapor–liquid–solid, SiO_2_ decomposition, SiO*_x_* nanowires

## Abstract

This work reports the formation of nanoflowers after annealing of Au/Ni bilayers deposited on SiO_2_/Si substrates. The cores of the nanoflowers consist of segregated Ni silicide and Au parts and are surrounded by SiO*_x_* branches. The SiO_2_ decomposition is activated at 1050 °C in a reducing atmosphere, and it can be enhanced more by Au compared to Ni. SiO gas from the decomposition of SiO_2_ and the active oxidation of Si is the source of Si for the growth of the SiO*_x_* branches of the nanoflowers. The concentration of SiO gas around the decomposition cavities is inhomogeneously distributed. Closer to the cavity border, the concentration of the Si sources is higher, and SiO*_x_* branches grow faster. Hence, nanoflowers present shorter and shorter branches as they are getting away from the border. However, such inhomogeneous SiO gas concentration is weakened in the sample with the highest Au concentration due to the strong ability of Au to enhance SiO_2_ decomposition, and nanoflowers with less difference in their branches can be observed across the whole sample.

## Introduction

Substantial efforts have been devoted to developing different kinds of nanofabrication methods during the past decades. For example, silicon oxide (SiO*_x_*) nanostructures can be grown by the catalyzing effect of Au nanoparticles based on the vapor–liquid–solid (VLS) mechanism [1–4]. Au–SiO*_x_* nanoflowers consisting of Au nanoparticles and surrounding SiO*_x_* nanowires (NWs) show a significant enhancement of the photoluminescence (PL) emission compared with pure SiO*_x_* NWs due to the coupling effect between the local surface plasmon resonance (LSPR) of Au nanoparticles and the PL emission of SiO*_x_* [2]. Similar Au–SiO*_x_* nanoflowers have also been obtained by depositing Au thin films on Si substrates with a thick silicon dioxide (SiO_2_) layer and subsequent rapid heating in reducing atmosphere. Here, the Si vapor source is silicon monoxide (SiO) gas produced by the decomposition of the SiO_2_ layer or the active oxidation of the Si substrate at higher temperatures in oxygen-deficient environment [3–4]. Another cost-effective nanofabrication method, thin film dewetting, driven by the reduction of the surface energy and the interface energy has also been profusely studied because it provides a straightforward and fast way to produce nanoparticles [5–7].

The research of thin film dewetting has been extended to bilayers and multilayers for the synthesis of multicomponent nanoparticles [8–16], like alloyed AuNi and AuAg nanoparticles produced by the solid-state dewetting of bilayers [17–25]. Apart from Au, both Ni and Ag can also catalyze the growth of NWs based on the VLS mechanism [26–28], and Si NWs have been grown using alloyed AuAg nanoparticles [29]. Au–SiO*_x_* nanoflowers have been fabricated by combining the dewetting of Au thin films and the growth of NWs based on the VLS mechanism [3–4]. This has inspired the possible fabrication of nanoflowers from multicomponent nanoparticles and surrounding SiO*_x_* NWs, on which, to date, there is no relevant research. The addition of the second element brings more tailorable properties and broadens the range of applications. For instance, alloyed AuAg nanoparticles show a tunable LSPR peak by changing the Au/Ag ratio [30]. Also, through the leaching of the less noble element, such as Ag in AuAg nanoparticles, dealloying can yield porous Au nanosponges with excellent optical properties [31–35]. Thus, it is interesting and significant to extend the previous research of Au–SiO*_x_* nanoflowers formed by an Au single layer to bilayers and explore potential fabrication parameters.

In the present work, nanoflowers made of a core nanoparticle and surrounding SiO*_x_* NWs are synthesized from annealing thin Au/Ni bilayers with three Au/Ni layer thickness ratios (15 nm/5 nm, 10 nm/10 nm, and 5 nm/15 nm, denoted 15Au5Ni, 10Au10Ni, and 5Au15Ni, respectively) deposited onto SiO_2_ (300 nm)/Si substrates. Au enhances the SiO_2_ decomposition rate stronger than Ni, leading to denser decomposition cavities observed in samples with thicker Au layer. Compared with previous works on Au–SiO*_x_* nanoflowers [3–4], an additional epitaxial NiSi_2_ structure can be found inside the cavities. The inhomogeneous distribution of SiO concentration outside the cavities enables the formation of nanoflowers with branches of changing length. The branches become shorter as they are getting far away from the border of the cavities. However, above inhomogeneity is decreased in the sample with the thickest Au layer due to the enhanced SiO_2_ decomposition and, consequently, the greater amount of produced SiO gas. Therefore, nanoflowers can be found only locally around the cavities in samples with smaller Au concentration, but they can be observed everywhere in the sample with the highest Au concentration.

## Results and Discussion

**Figure 1:**
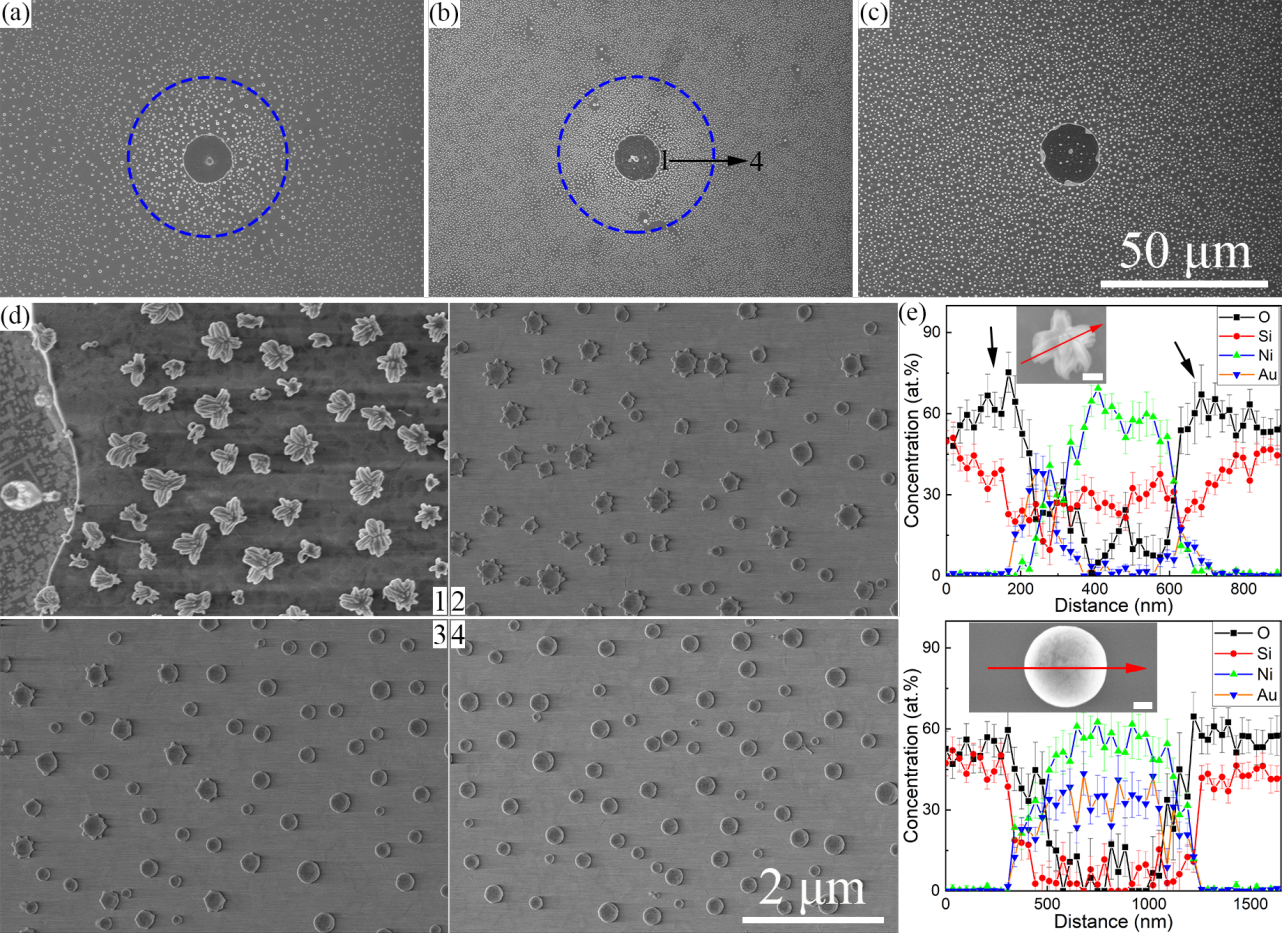
Morphology around decomposed areas (a–c). Distribution and composition of nanoflowers and particles outside the decomposition cavity in 10Au10Ni, respectively (d, e). Images (a–c) show 5Au15Ni, 10Au10Ni and 15Au5Ni, respectively. The dotted circles in (a, b) show the boundary of nanoflowers and particles. Images 1–4 in (d) show areas increasingly further away from the border of the cavity in 10Au10Ni, as marked in (b). The scale bar in (c) is also valid for (a, b), and the scales of the four images in (d) are the same. The scale bars of the inset in (e) are 200 nm.

Au/Ni bilayers with three thickness combinations were deposited on SiO_2_/Si substrates. After annealing at 1050 °C for 1 min in forming gas (mixture of Ar and H_2_), scattered spots (Supporting Information File 1, Figure S1) can be found on the surface. The enlarged insets present the circular feature of those spots and their height distributions indicate that circular areas are below the substrate surface. Hence, they will be referred to as cavities below. The enlarged view of the morphologies of the circular spots and the structure details outside the cavities are shown in Figure 1. Flower-like structures, called nanoflowers below, and particles with smooth surfaces can be observed in 5Au15Ni and 10Au10Ni. However, only nanoflowers are found in 15Au5Ni. The nanoflowers exhibit different morphologies as shown in Figure 1d. The length and number of their branches decrease with increasing distance from the border of the cavity in 10Au10Ni. The EDS result of one nanoflower is also shown in Figure 1e. The core of the nanoflower is mainly composed of Au, Ni, and Si while its branch parts show a much faster increase in O concentration than that of Si, indicating the possible formation of SiO*_x_* branches. To further detail the composition, EDS results were measured on the tilted morphology showing larger areas of the branch part (Supporting Information File 1, Figure S2). A similar concentration of O and Si corresponding to the substrate agrees well with Figure 1e. However, a much higher O concentration than that of Si corresponding to the branch part proves the possibility of SiO*_x_* branches again. Also, both Au and Ni show negligible concentrations, which means that EDS measured only substrate and branch parts. In accordance with previous works [3–4], the nanoflowers can be identified as heterostructures with a core particle and surrounding SiO*_x_* nanowires. The core particle is made of segregated Ni silicide and Au, which can be proved by their heterogeneous distributions in Figure 1e. Similar results of the other two samples are summarized in Supporting Information File 1, Figure S3 and Figure S4.

The formation of the circular cavities can be attributed to the decomposition of the SiO_2_ layer at high temperature in reducing atmosphere. It has been reported after the annealing of Au thin films deposited on SiO_2_/Si substrates with different thicknesses of the SiO_2_ layer [3–4,36]. The active oxidation of Si also occurs once the Si substrate is exposed [2–3,37], which can be proved by the calculated oxygen partial pressure (Supporting Information File 1) and the much greater average depths of cavities (more than 600 nm) compared with the thickness of the SiO_2_ layer (300 nm). Besides, the number of visible spots increases with Au thickness as indicated by the numbers in Supporting Information File 1, Figure S1. Metallic elements, such as Au and Ni, can diffuse to the Si/SiO_2_ interface and enhance the decomposition rate there [38–40]. Hence, increasing decomposed areas with the thicker Au layer means that Au enhances the decomposition of SiO_2_ more than Ni.

**Figure 2:**
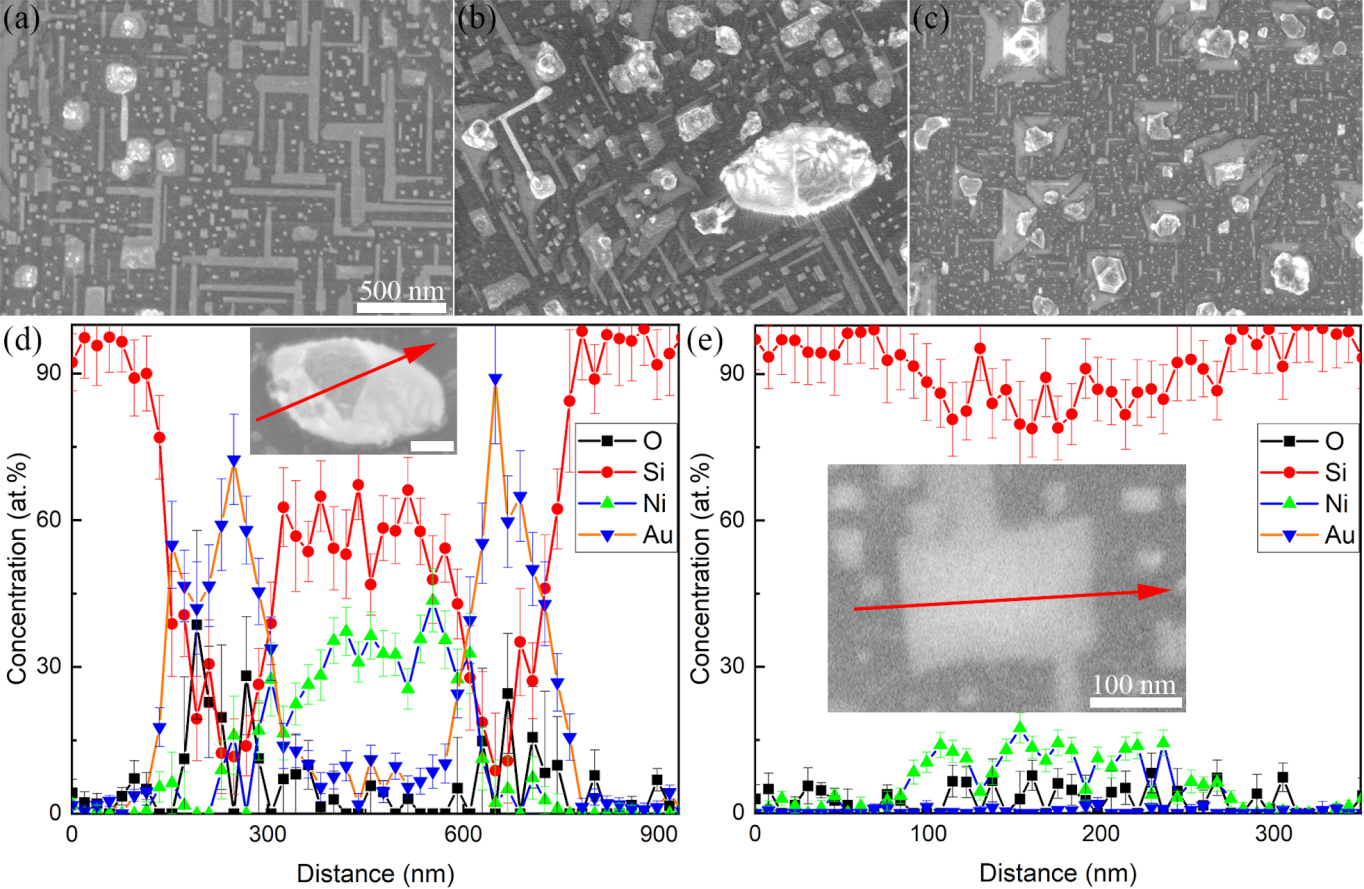
(a–c) Morphology inside the decomposition cavities. (d) Composition of the particle and (e) the line in 10Au10Ni. Images (a–c) correspond to 5Au15Ni, 10Au10Ni and 15Au5Ni, respectively. The scale bar in (a) is also valid for (b, c), and the scale bar of the insets in (d) is 200 nm.

Completely different structures can be observed inside the decomposed areas, as shown in Figure 2. There are mainly two shapes of microstructures, namely particles and lines. The particles present bright and dark parts. The bright areas should be rich in Au based on the material contrast, and the EDS results also indicate the high Au content in Figure 2. The dark areas consist of more Si and Ni in 5Au15Ni and 10Au10Ni but less Ni in 15Au5Ni, which has the lowest Ni concentration (Supporting Information File 1, Figure S5). The line structures show epitaxial self-assembly growth and their EDS results show a comparatively high content of Ni apart from Si, which may partially come from the substrate. Considering previous works in which the line structures were absent when only Au thin films were deposited [3–4], the existence of such epitaxial line structures should be highly related to the addition of Ni by depositing Au/Ni bilayers. This can also be proved by the plateau of Ni in EDS results of line structures. A number of works about self-assembled epitaxial Ni silicide have been published [41–46], and some works pointed out that the Ni_2_Si phase formed first, followed by NiSi and NiSi_2_ after annealing [47–49]. Generally, NiSi_2_ forms above 600 °C [42–45,48]. Therefore, the self-assembled epitaxial line structures in this work are supposed to be NiSi_2_.

**Figure 3:**
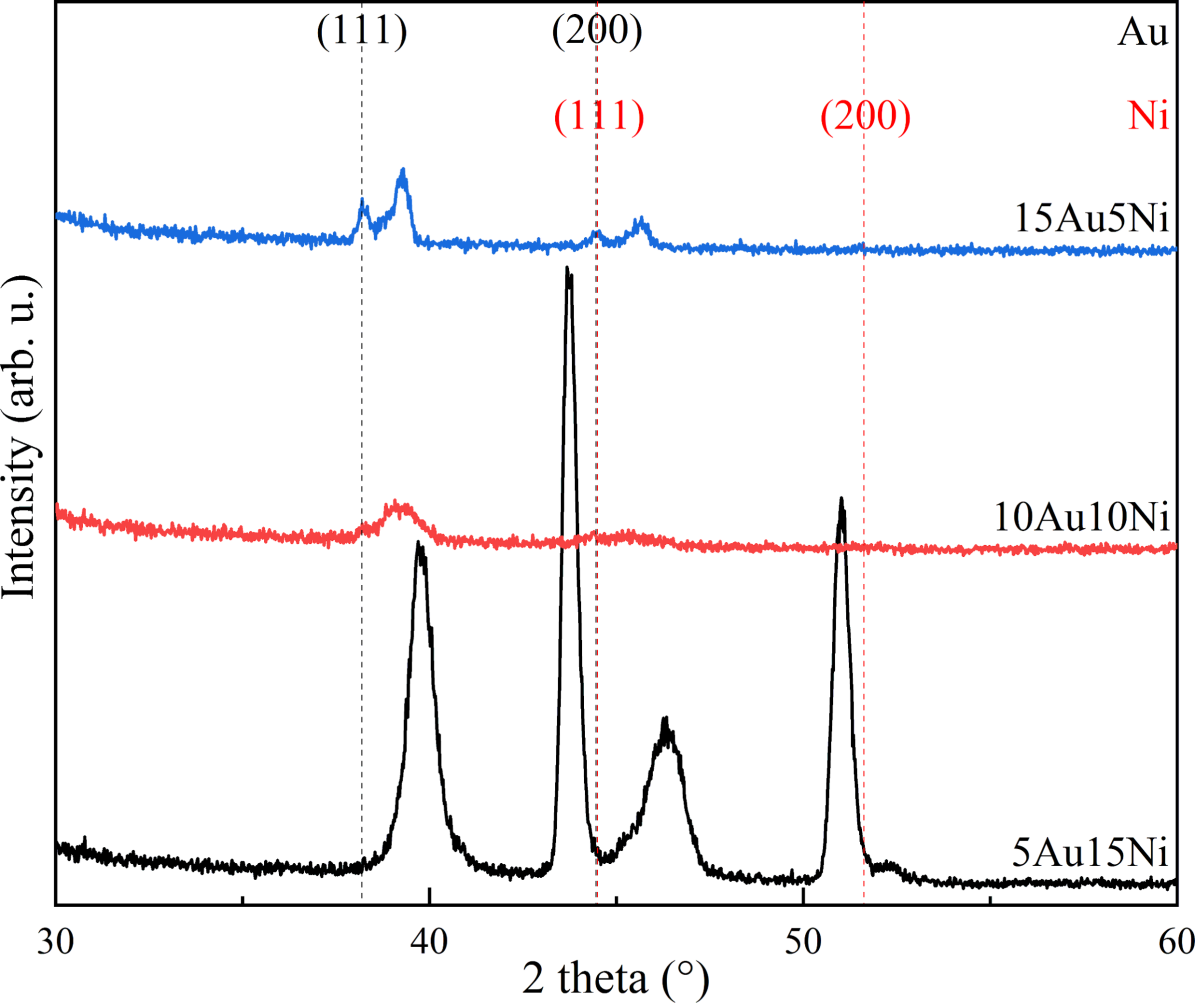
XRD patterns of the dewetted systems after annealing at 1050 °C. The standard data of Au (PDF 03-065-2870) and Ni (PDF 03-065-0380) are listed.

XRD patterns are shown in Figure 3. Most reflexes show clear deviations from the reported positions of Ni silicide (Supporting Information File 1, Figure S6). The absence of Ni silicide reflexes may be attributed to the low concentrations of the Ni silicide phases, because the line structures are only observed inside the cavities, which only account for a very small percentage of the whole sample surface. The reflex positions of pure elemental Au and Ni are marked in Figure 3. Both Au and Ni are mixed to a great extent after annealing, which is confirmed by the main peak shifts between the positions of pure Au and Ni. However, the mixing is incomplete because there are still small peaks of pure Au and Ni. The partial mixing can also be evidenced by the multiple reflexes between the positions of pure Au and Ni since only one main reflex should be observed when the two elements are completely mixed [20,23,25]. The annealing temperatures are above the miscibility gap [23,50]. Thus, the partial mixing comes from the phase separation of Au and Ni during cooling [25].

**Figure 4:**
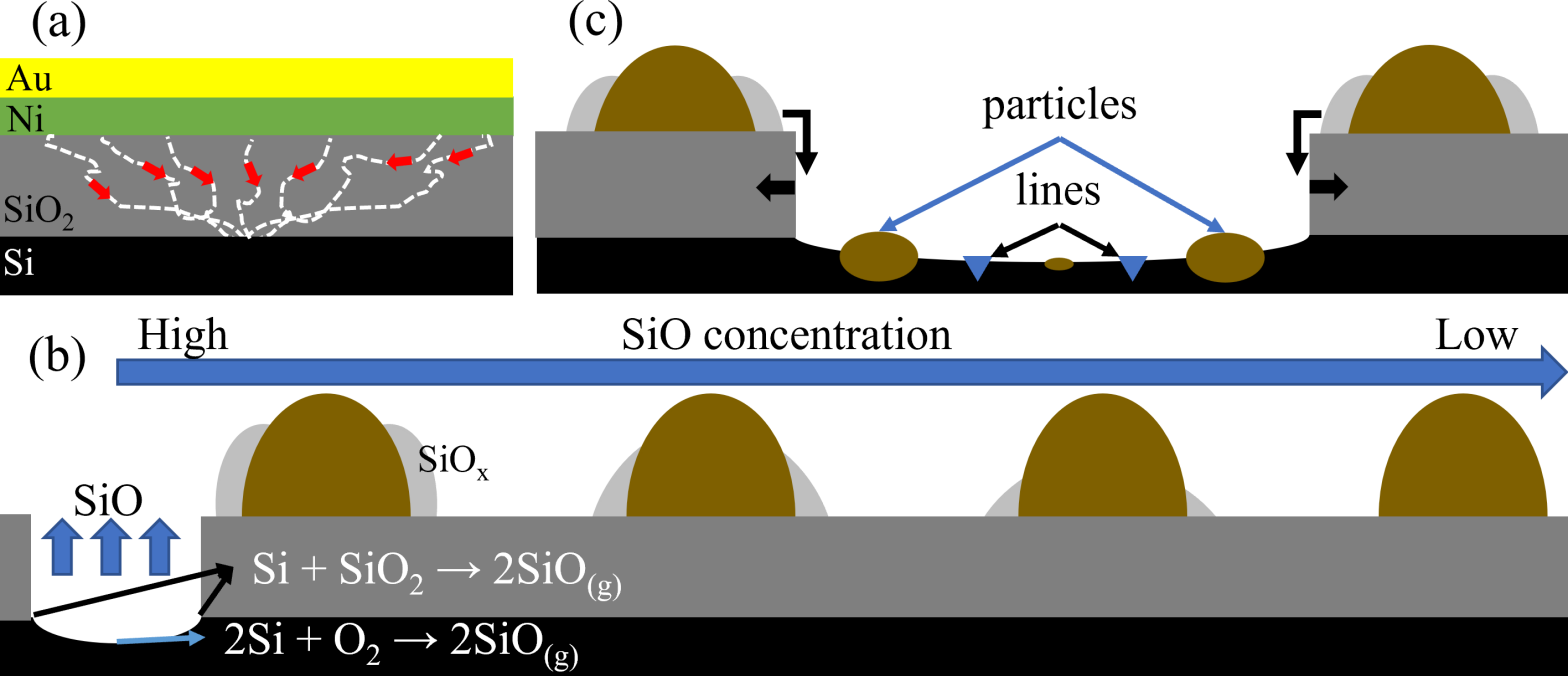
Formation mechanisms at elevated temperatures. (a) As-deposited bilayers and Au/Ni diffusion along nanochannels (dashed lines) in the SiO_2_ layer. All dashed lines pointing to the common point at the SiO_2_/Si interface aim to highlight the enhancement of SiO_2_ decomposition by the thin films. (b) SiO concentration gradient outside the decomposition cavity and cross sections of nanoflowers with changing branch length. (c) Cross sections of particles and epitaxial line structures inside the decomposition cavity.

According to the results presented above, in Figure 4, we propose the following processes to explain the formation of nanoflowers with changing size in their branches outside the decomposed areas as well as the particles and epitaxial line structures inside the decomposed areas. Similar to previous works [3–4], dewetting of the Au/Ni bilayers and diffusion of Au and Ni atoms from the bilayers to the SiO_2_/Si interface begin at high temperatures. Simultaneously, decomposition is initiated at the SiO_2_/Si interface, and it can be strengthened by the diffused Au and Ni atoms to finally form the decomposition cavities. The active oxidation of Si also happens once the Si substrate is exposed [2–3,37]. Both decomposition and active oxidation can produce volatile SiO gas as the Si vapor source for the formation of SiO*_x_* NWs based on VLS mechanism [2,26–27,51–52]. Several NWs nucleate and then grow around particles because they are large enough to provide several nucleation sites [2–3], leading to the shape of flowers. Since SiO vapor can be only formed in the cavities, there is a non-uniform distribution of SiO gas concentration around the cavities. Namely, the closer to the cavities, the higher the concentration of the SiO gas, as shown in Figure 4b. This inhomogeneous distribution of the growth source leads to the different growth rates of nanoflowers in the area around the cavities. Basically, higher source concentrations enable higher growth speeds. This is why the particles close to the cavities grow into nanoflowers with much longer branches, whereas further away only small SiO*_x_* NWs or even no NWs are formed (Figure 1d). Similar uneven distributions of the Si source have been reported [2]. There are more cavities in 15Au5Ni than in the other two samples (Supporting Information File 1, Figure S1), meaning more SiO gas is produced. Thus, the inhomogeneity of the Si source is reduced and particles far away can also grow into nanoflowers (Supporting Information File 1, Figure S4). A much weaker inhomogeneity of the Si source has also been observed in the case of Au single layer when using similar annealing parameters [3], which further proves the higher ability of Au, compared to Ni, to enhance the SiO_2_ decomposition.

The cavities keep growing laterally after piercing vertically the SiO_2_ layer and exposing the Si substrate [39,53–54]. Then, structures around the border of cavities will drop inside and get in contact with the exposed Si substrate, as marked in Figure 4c. The outer SiO*_x_* NWs can be decomposed by the Si substrate, and the core particle consisting of Au and Ni can get in direct contact with the substrate. Thus, Au–Si droplets and Ni silicide can form due to the easy interdiffusion of Au, Ni, and Si. Au/Si phase separation occurs during cooling [3,55], and Ni silicide may remain stable down to room temperature [41–46], finally forming particles with two contrasts. Besides, Ni may also diffuse into the Si substrate, leading to the formation of the Ni silicide, and a cross-sectional view of Ni silicide is given in Figure 4c based on reported works [44,48,56]. The elongation process of the symmetric NiSi_2_ clusters is mainly governed by the growth kinetics [44,57–58].

## Conclusion

In the present work, nanoflowers with a core particle and surrounding SiO*_x_* NWs have been produced on a SiO_2_ (300 nm)/Si substrate after a rapid heat treatment. The core particle consists of segregated Ni silicide and Au. A high temperature of 1050 °C can activate the decomposition of SiO_2_. Together with the subsequent active oxidation of Si, it provides the volatile SiO gas for the growth of SiO*_x_* NWs. Au has a greater ability to enhance the SiO_2_ decomposition than Ni, which leads to the formation of more cavities in the sample with higher Au concentration. Two kinds of structures are formed inside the decomposition cavities, that is, particles showing two contrasts and lines presenting epitaxial growth. The non-uniform distribution of SiO gas concentration around the decomposition cavities leads to the different growth rates of SiO*_x_* branches in the nanoflowers based on their distance to the border of the cavities. The closer to the border, the higher the SiO concentration around the nanoflowers and the faster the growth speed of their SiO*_x_* branches, forming nanoflowers with shorter and shorter branches as their locations get farther away from the border. Therefore, nanoflowers can be only observed locally around decomposition cavities, and only isolated particles with smooth surface can be found within the areas beyond a certain distance from the cavity border. However, this inhomogeneity is relatively weak in the sample with the highest Au concentration, because of the greater ability of Au to enhance SiO_2_ decomposition leading to more volatile SiO and reducing the concentration gradient. As a result, although nanoflowers still present longer branches near the cavities, they can be observed across the whole sample rather than only in local areas in samples with less Au.

## Experimental

Four-inch single-side polished p-type (100)-oriented Si-wafers were used. A 300 nm oxide layer was thermally grown to prevent interactions between Si and the subsequently deposited layer materials, and after that, the wafer was cut into small squares of approximately 1 cm × 1 cm. After cleaning in acetone, isopropanol, and deionized water and drying with nitrogen gas, the small pieces were ready for thin film deposition. Metallic bilayers of Au and Ni of three different thickness ratios and a total thickness of 20 nm were deposited onto the SiO_2_/Si substrate by electron beam evaporation (CS400ES, VON ARDENNE) at a working pressure of 1 × 10^−6^ mbar. The Au layer was always deposited after the Ni layer to prevent the oxidation of the Ni layer. The bilayer thicknesses of different systems were 15 nm Au/5 nm Ni, 10 nm Au/10 nm Ni and 5 nm Au/15 nm Ni, and the thickness of each layer was controlled by a quartz balance during the deposition. Thermal annealing was carried out in a rapid thermal processing (RTP, Jipelec Jetstar 100) furnace. First, the chamber was evacuated and purged with Ar three times at room temperature, then a flow of forming gas of Ar + H_2_ (volume ratio 30:1) was kept till the end of the experiment. The temperature was ramped up to 300 °C in 20 s, where it was hold for 30 s. After that, the samples were rapidly heated to 1050 °C within 15 s and then held at this temperature for 1 min before cooling down. One fresh sample was heated for each treatment. Each system was labeled according to its composition. For example, the sample 15 nm Au/5 nm Ni annealed at 1050 °C was named 15Au5Ni.

The morphology was imaged by optical microscopy (OM, Zeiss Axiotech) and high-resolution scanning electron microscopy (HR-SEM, Hitachi S-4800) equipped with energy-dispersive X-ray spectroscopy (EDS, Thermo Scientific). The SEM images were recorded by using mixed signals from secondary electrons and backscattered electrons (BSE) to minimize charging effects due to the bad electrical conductivity of the SiO_2_ layer. In addition, the composition information related to the *Z*-contrast was obtained by the BSE detector because the areas rich in elements with higher atomic numbers show brighter contrasts. EDS measurements were performed to obtain the element distribution in the target areas. X-ray diffraction (XRD, Siemens D-5000) analyses were conducted in Bragg–Brentano mode using Cu Kα irradiation at 40 kV. The height distribution of the areas of interest was measured by laser scanning microscopy (LSM, Olympus LEXT 4100).

## Supporting Information

File 1 Additional OM, LSM, SEM, EDS and XRD measurements.
