# Supplementary material for: Formation of nanoflowers: Au and Ni silicide cores surrounded by SiOx branches
Source: Beilstein J Nanotechnol. 2023 Jan 20;14:133–40. doi: 10.3762/bjnano.14.14 (PMC9874233; doi:10.3762/bjnano.14.14)
Supplement: File 1 — Additional OM, LSM, SEM, EDS and XRD measurements. [file Beilstein_J_Nanotechnol-14-133-s001.pdf]

BEILSTEIN JOURNAL OF NANOTECHNOLOGY

Supporting Information

for

Formation of nanoflowers: Au and Ni silicide cores surrounded by SiO_x branches

Feitao Li, Siyao Wan, Dong Wang and Peter Schaaf

Beilstein J. Nanotechnol. **2023,** *14,* 133–140. doi:10.3762/bjnano.14.14

Additional OM, LSM, SEM, EDS and XRD measurements

License and Terms: This is a supporting information file under the terms of the Creative Commons Attribution License (https://creativecommons.org/licenses/by/4.0). Please note that the reuse, redistribution and reproduction in particular requires that the author(s) and source are credited and that individual graphics may be subject to special legal provisions.
The license is subject to the *Beilstein Journal of Nanotechnology* terms and conditions: (https://www.beilstein-journals.org/bjnano/terms)

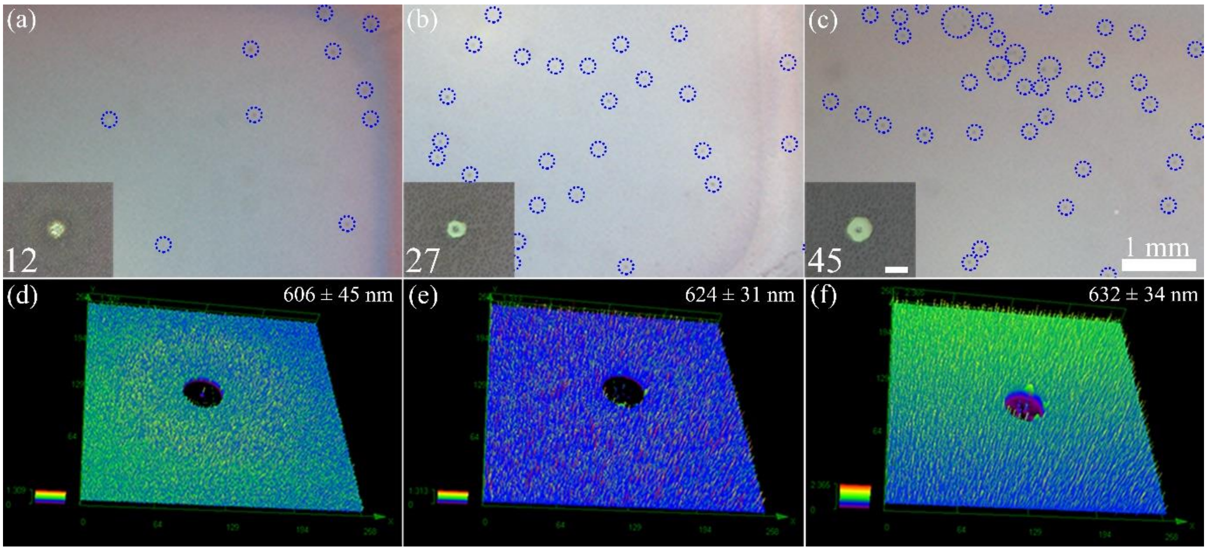

Figure S1: (a-c) OM images. (d-f) LSM results. Images (a, d), (b, e), (c, f) correspond to 5Au15Ni, 10Au10Ni and 15Au5Ni, respectively. Insets in (a-c) show the magnified morphologies of partially marked spots. The number of spots from each image is given in the bottom left corner. The scale bars in (c) are also valid for (a, b) and the scale bar of the inset in (c) is 10 μm. The average depth of each circular area is also shown at the upper right corner in d-f.

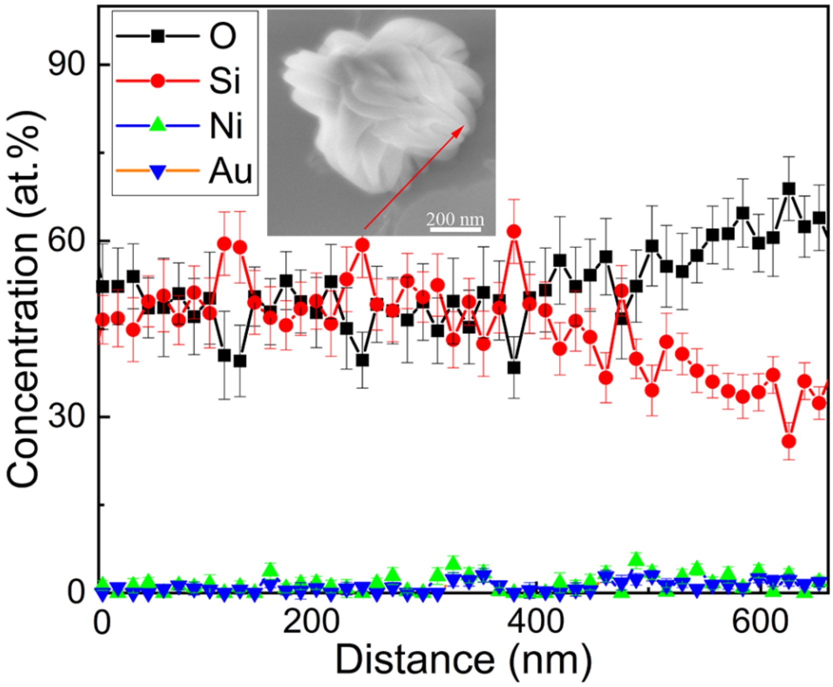

Figure S2: Composition of nanoflowers in 10Au10Ni from the tilted SEM view.

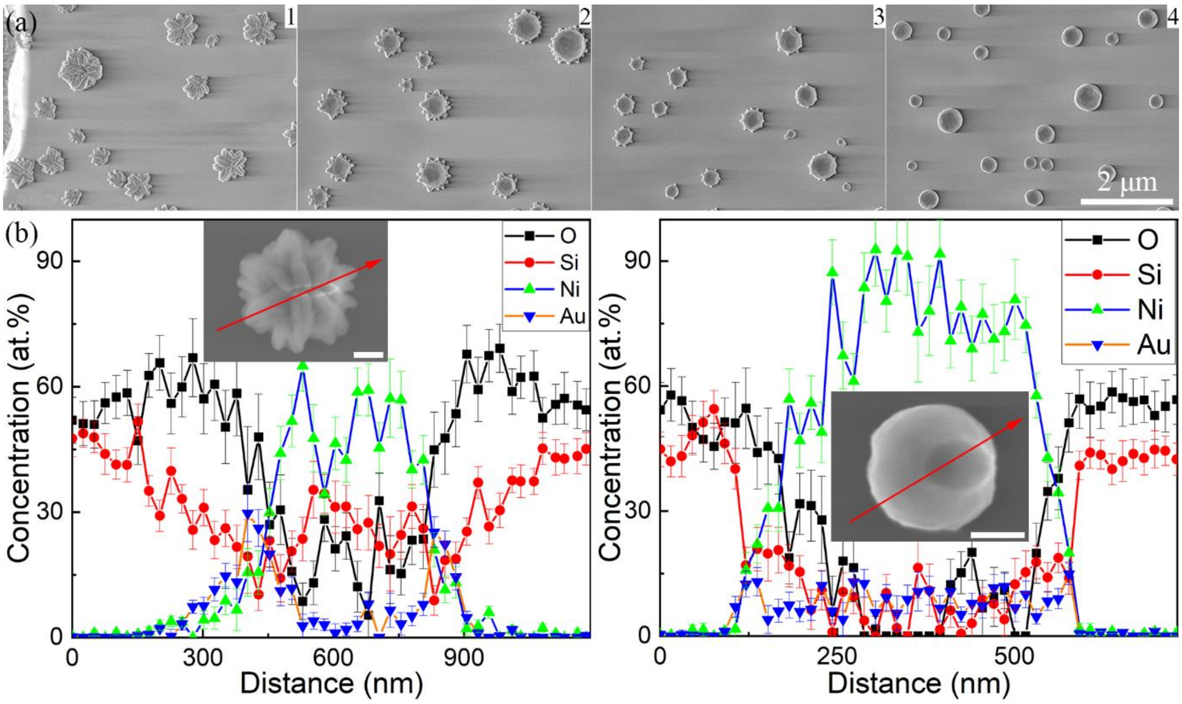

Figure S3: (a, b) Distribution and composition of nanoflowers and particles outside the decomposed cavity in 5Au15Ni, respectively. Images 1-4 in (a) show the areas becoming far and far from the border of the cavity in 5Au15Ni. The scale bars of four images in (a) are the same and the scale bar of the inset in (e) is 200 nm.

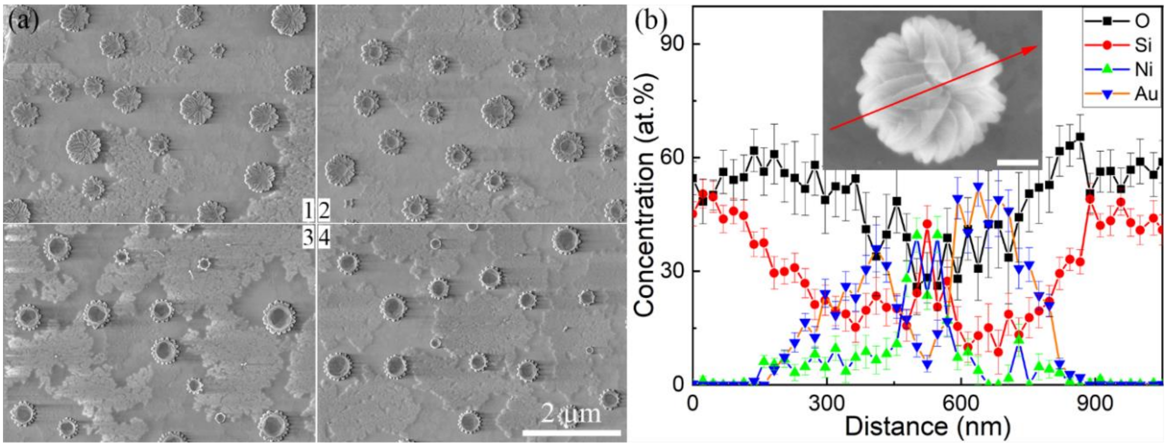

Figure S4: (a, b) Distribution and composition of nanoflowers outside the decomposed cavity in 15Au5Ni, respectively. Images 1-4 in (a) show the areas becoming far and far from the border of the cavity in 15Au5Ni. The scale bars of four images in (a) are the same and the scale bar of the inset in (e) is 200 nm.

Calculation of oxygen partial pressure:

In the present work, the purities of used Ar and H2 are 6.0N (99.9999%) and 5.0N (99.999%), respectively. The volume ratio of Ar and H_2 is 30:1. The partial oxygen pressure should be related to the residual gas and to the impurities of the Ar and H_2, and it can be estimated as follow:

$$\frac{V_{O2}}{V_{total}} = \frac{p_{O2}}{p_{total}}$$

As we use Ar and H_2 in a 30:1 volume ratio, we can consider that we have 31 parts of volume. In the case of Ar, a purity of 99.9999% leads to 0.0001% of impurities, and the same applies to H_2. Assuming that the impurities of the gases have the same composition than air, with 21% oxygen, the partial pressure of oxygen can be estimated as:

$$p_{O2} = p_{total} \times 21\% \times \frac{(V_{Ar} \cdot \text{impurities}_{Ar} + V_{H2} \cdot \text{impurities}_{H2})}{V_{total}} = \frac{\frac{21}{100}(30 \cdot 10^{-6} + 1 \cdot 10^{-5})}{31} = 0.27 \text{ ppm}$$

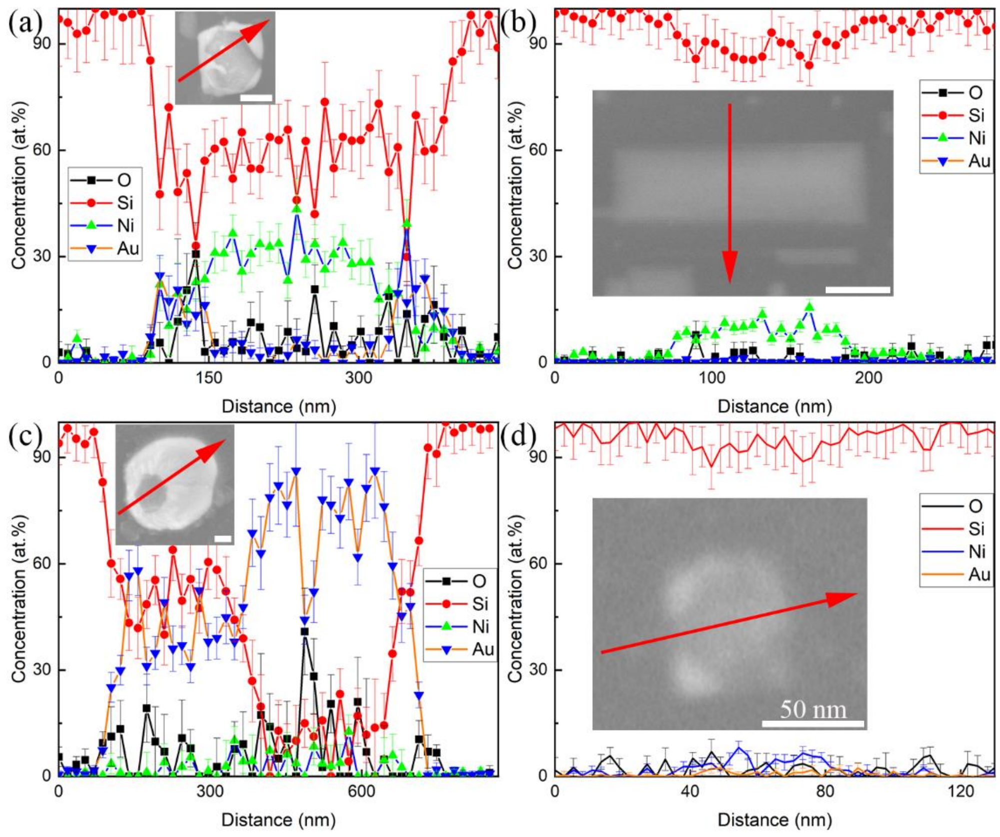

Figure S5: EDS results in cavities. (a, b) 5Au15Ni. (c, d) 15Au5Ni. Particles (a, c) and lines (b, d). Scale bars of other insets: 100 nm.

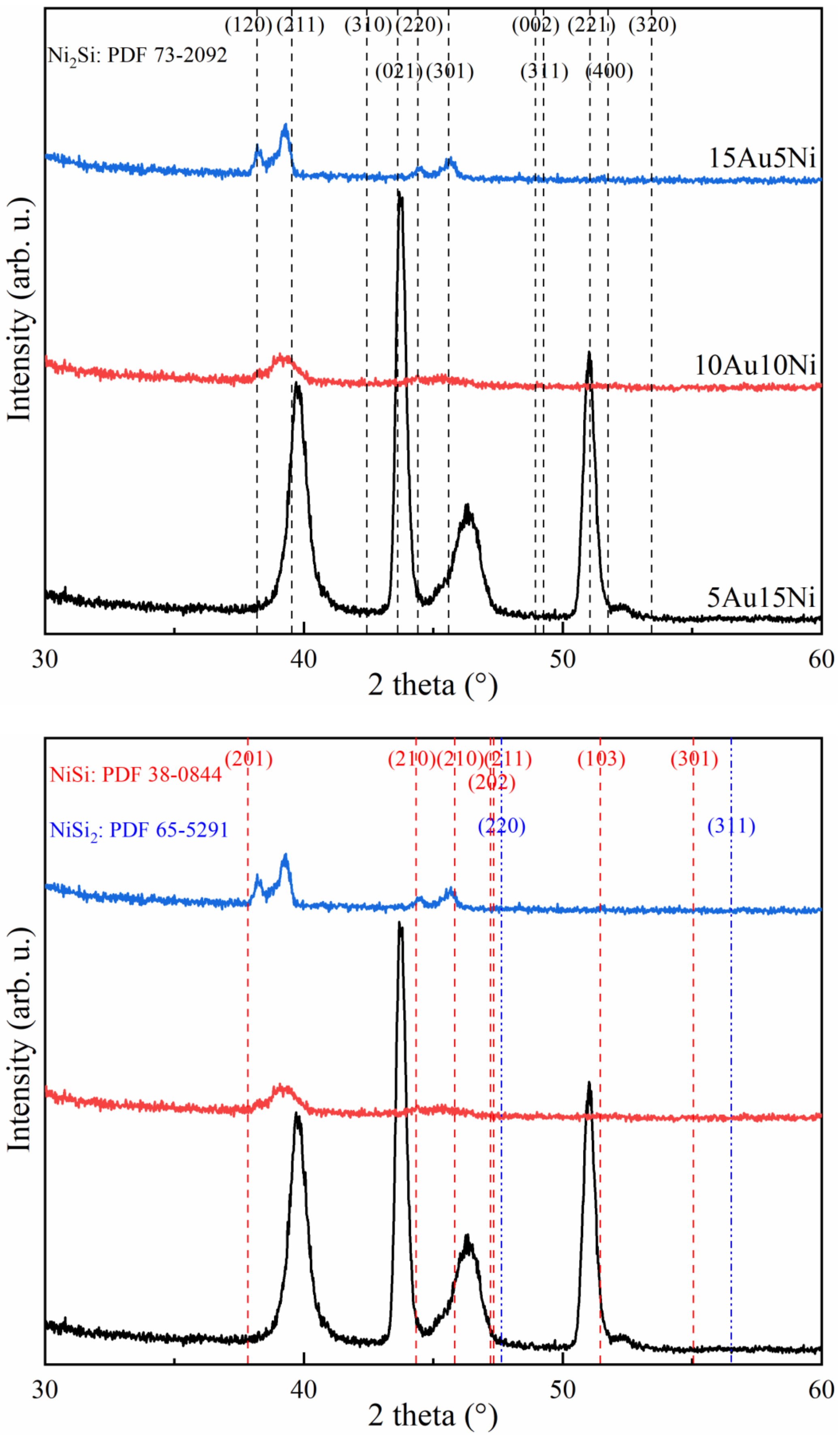

Figure S6: XRD results of Figure 3 with peak indication of three Ni silicide phases.
